# A functional anatomical shift from the lateral frontal pole to dorsolateral prefrontal cortex in emotion action control underpins elevated levels of anxiety: partial replication and generalization of Bramson *et al*., 2023

**DOI:** 10.1093/psyrad/kkaf009

**Published:** 2025-04-28

**Authors:** Qian Zhuang, Shuxia Yao, Lei Xu, Shuaiyu Chen, Jialin Li, Xiaoxiao Zheng, Meina Fu, Keith M Kendrick, Benjamin Becker

**Affiliations:** Center for Cognition and Brain Disorders, The Affiliated Hospital of Hangzhou Normal University, Hangzhou Normal University, Zhejiang Province, Hangzhou 311121, China; The Clinical Hospital of Chengdu Brain Science Institute, MOE Key Laboratory for Neuroinformation, Center for Information in Medicine, School of Life Science and Technology, University of Electronic Science and Technology of China, Chengdu 611731, China; Institute of Brain and Psychological Sciences, Sichuan Normal University, Chengdu 610066, China; Center for Cognition and Brain Disorders, The Affiliated Hospital of Hangzhou Normal University, Hangzhou Normal University, Zhejiang Province, Hangzhou 311121, China; The Clinical Hospital of Chengdu Brain Science Institute, MOE Key Laboratory for Neuroinformation, Center for Information in Medicine, School of Life Science and Technology, University of Electronic Science and Technology of China, Chengdu 611731, China; Brain Cognition and Brain Disease Institute (BCBDI), Shenzhen Institute of Advanced Technology, Chinese Academy of Sciences, Shenzhen 518055, China; The Clinical Hospital of Chengdu Brain Science Institute, MOE Key Laboratory for Neuroinformation, Center for Information in Medicine, School of Life Science and Technology, University of Electronic Science and Technology of China, Chengdu 611731, China; The Clinical Hospital of Chengdu Brain Science Institute, MOE Key Laboratory for Neuroinformation, Center for Information in Medicine, School of Life Science and Technology, University of Electronic Science and Technology of China, Chengdu 611731, China; Institute of Science and Technology for Brain-Inspired Intelligence, Fudan University, Shanghai 200433, China; State Key Laboratory of Brain and Cognitive Sciences, The University of Hong Kong, Hong Kong, China; Department of Psychology, The University of Hong Kong, Hong Kong 999077, China

**Keywords:** emotion, inhibition, anxiety, frontal pole, DLPFC, Go/NoGo task

## Abstract

**Background:**

Emotion control represents a promising intervention target for mental disorders. In a recent study Bramson et al. (2023) demonstrate a functional–anatomical shift from the lateral frontal pole (FPl) to the dorsolateral prefrontal cortex (DLPFC) in anxious individuals during emotional action control. However, findings of neuroimaging experiments are often limited regarding generalizability and reproducibility. The present study examined the robustness of the reported functional shift across samples, cultures and paradigms.

**Methods:**

We capitalized on large-scale task fMRI data (*n* = 250 participants) using an affective linguistic Go/NoGo paradigm to examine the anxiety-related shift between FPl and DLPFC during emotional action control. Additionally, context-dependent functional connectivity analyses were employed to examine anxiety-related differences and associations on the network level.

**Results:**

Non-anxious individuals engaged the left FPl while highly anxious individuals specifically recruited the DLPFC, but non-significant between-group differences were found (see also Bramson et al.). The secondary analyses revealed moderate evidence for the absence of left FPl activation in the high-anxious as well as for left DLPFC activation in the non-anxious group. Additionally, trait anxiety scores were positively correlated with left DLPFC activity but negatively correlated with left FPl activity across groups. Furthermore, we found a context-specific connectivity shift between the subgenual anterior cingulate cortex (sgACC) with the FPl and DLPFC specifically in highly anxious individuals.

**Conclusion:**

The results partially confirmed the anxiety-related shift as reported by Bramson and colleagues across paradigms and samples. The findings provide further support for the functional shift in anxiety and can inform target-based interventions of persistent emotional control deficits in anxiety disorders.

## Introduction

Flexible control over emotional behavior is vital for mental health and represents a promising target for novel interventions for mental disorders (e.g. Etkin *et al*., 2015; Feng *et al*., 2018). Accumulating evidence from task-based neuroimaging studies indicates a key role of the lateral frontal pole (FPl) and its connections with other cortical and subcortical systems in regulating emotional action tendencies (Tyborowska *et al*., 2016, 2024; Fonzo *et al*., 2017; Bramson *et al*., 2023). In particular, a recent study from Bramson *et al*. (2023) employed a multi-modal neuroimaging approach to demonstrate a functional–anatomical shift in a sample of anxious individuals, such that high-anxious individuals engaged the dorsolateral prefrontal cortex (DLPFC), while non-anxious individuals engaged the FPl during control of emotional action tendencies. While these findings might represent a venue for interventions targeting persistent emotional control deficits in anxiety disorders (Meijer *et al*., 2023), conventional neuroimaging strategies are often limited with respect to generalizability and reproducibility (e.g. Poldrack *et al*., 2017; Zhou *et al*., 2022; Gan *et al*., 2024).

**Figure 1:**
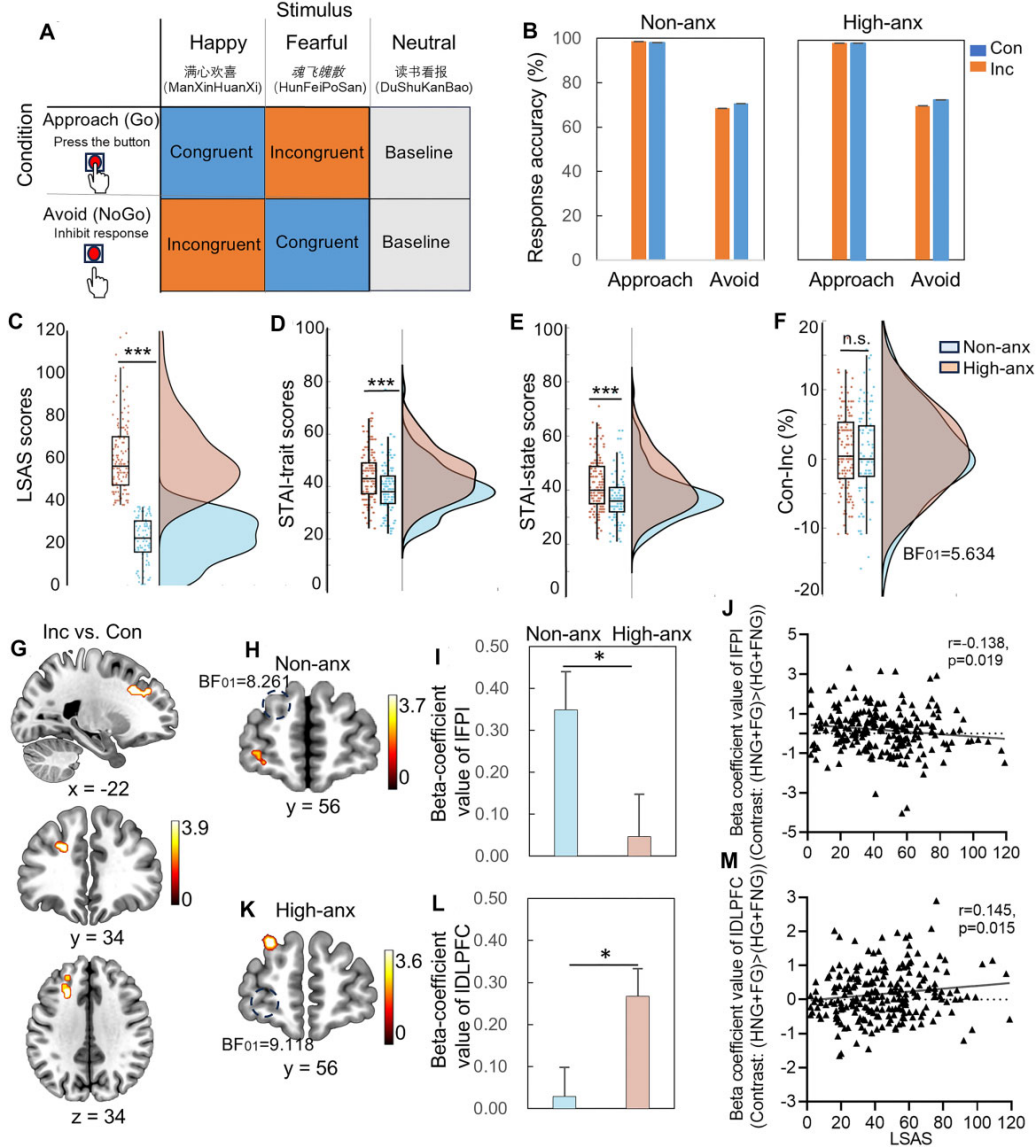
Behavioral and neural-congruence effect during emotional behavioral control with the affective Go/NoGo paradigm. (A) Congruent–incongruent effect as modeled by the affective Go/NoGo task used in the current study to delineate the interaction between emotion and behavioral factors. (B) Mixed ANOVA on response accuracy with congruence (incongruent/congruent) × action (approach/avoid) × group (high-/non-anxious) as variables showed a main effect of congruence (*F*_(1,225) _= 6.174, *P* = 0.014) and action (*F*_(1,225) _= 692.071, *P* < 0.001). Neither the interaction effect between congruence × action × group (*F*_(1,225) _= 0.001, *P* = 0.970) nor the interaction between congruence × group (*F*_(1,225) _= 0.415, *P* = 0.520) was significant with (F) the Bayesian t-test showing moderate evidence for the absence of a group difference on the behavioral congruence effect (BF_01 _= 5.634). Group differences on LSAS (C), TAI (D) and SAI (E) with LSAS 38 as the cutoff score. (G) Neural-congruence effect across groups showed a significant activation in left DLPFC after small volume correction. (H and K) Examination of the anxiety groups with one-sample tests [based on the contrast: (HNG + FG) > (HG + FNG)] confirmed that the non-anxious group mainly engaged the left FPl [small volume correction (SVC), *Z* = 3.54, *P*_FWE _= 0.039, voxels = 12, *x*/*y*/*z*: −30, 60, −3), while the high-anxious group specifically recruited the DLPFC (SVC, *Z* = 3.47, *P*_FWE _= 0.048, voxels = 13, *x*/*y*/*z*: −27, 42, 36; threshold: SVC in combination with peak-level FWE corrected at *P* < 0.05). (I and L) Parameter estimates [contrast: (HNG + FG) > (HG + FNG)] extracted by the activated region confirmed group differences on the BOLD signal of left FPl and DLPFC with stronger recruitment of left FPl and DLPFC in the non-anxious group and high-anxious group, respectively. (J and M) Brain BOLD signal [contrast: (HNG + FG) > (HG + FNG)] and trait anxiety association analyses showed a significant negative association between left FPl activation and LSAS scores (*r* = 0.138, *P* = 0.019) and a positive association between left DLPFC and LSAS scores (*r* = 0.145, *P* = 0.015) across groups (following a Bonferroni correction *P* < 0.05/4 = 0.0125, trend-to-significant) but not for STAI scores (all *P* > 0.226). Note: BOLD, blood oxygen level-dependent; Con, congruent; DLPFC, dorsolateral prefrontal cortex; FG, Fearful Go; FNG, Fearful NoGo; FPl, lateral frontal pole; HG, Happy Go; High-anx: high-anxious group; HNG, Happy NoGo; Inc, incongruent; LSAS, Liebowitz Social Anxiety Scale; non-anx, non-anxious group; STAI, State-Trait Anxiety Inventory. *P < 0.05, ***P < 0.001, n.s., non-significant.

A functional magnetic resonance imaging (fMRI) study from our team (Zhuang *et al*., 2021, 2023) in 250 healthy individuals used a motor control task (affective linguistic Go/NoGo paradigm, Fig. 1A) to examine the robustness of the reported associations with social anxiety across samples, cultures, and paradigms. The large dataset allowed us to split the sample into high- and non-anxious groups and the paradigm allowed us to model emotional action control as the capability to override the automatic action tendencies evoked by emotional words in affect-incongruent conditions [Happy NoGo (HNG) and Fearful Go (FG) trials] as compared to congruent conditions [Happy Go (HG) and Fearful NoGo (FNG) trials] during the emotion and inhibitory control interactions according to a well-established Pavlovian bias framework (Guitart-Masip *et al*., 2012, 2014). Additionally, based on previous studies showing the contribution of intrinsic and emotion-specific network-level changes to elevated anxiety (Xu *et al*., 2021; Chen *et al*., 2023) and the key role of interactions between the subgenual anterior cingulate cortex (sgACC) with medial and lateral PFC regions in emotion-related cognitive action control (Lapate *et al*., 2022), we examined whether the network-level interaction of these regions varies as a function of anxiety levels.

## Materials and methods

### Participants and task setting

In total, 250 healthy, right-handed Chinese students were enrolled in the current study after given written informed consent and performed an affective Go/NoGo task with concurrent fMRI (for details see Zhuang *et al*., 2021, 2023). After quality control, *n* = 227 participants were divided into high- (*n *= 127) and non-anxious group (*n* = 100, see Supplementary Information) using the Liebowitz Social Anxiety Scale (LSAS, Mennin *et al*., 2002; Fig. 1C) with 38 as the cutoff score (based on He & Zhang, 2004, reporting both sensitivity and specificity >80% in a Chinese population). Groups were further validated with State-Trait Anxiety Inventory-trait scale (STAI-trait, *P* < 0.001, Fig. 1D) and state anxiety differences (*P* < 0.001, Fig. 1E, Spielberger *et al*., 1983).

During the task, positive (happy), negative (fearful), and neutral words (word length = 4, words frequency matched) were used as task stimuli. Participants were instructed to respond according to the font of the words regardless of the meaning, such as pressing the button when the words were presented as normal font (i.e. Go trials, approach condition), while withdrawing the response when the words were occasionally in italic font (i.e. NoGo trials, avoid condition). Here, while happy Go trials and fearful NoGo trials represented approaching- and avoiding-congruent conditions, respectively, fearful Go and happy NoGo trials represented approaching- and avoiding-incongruent trials. The study was approved by the local ethics committee and in accordance with the latest revision of the *Declaration of Helsinki*.

### Data acquisition and analysis

#### Behavioral data analysis

For response accuracy, a mixed three-way ANOVA with congruence (incongruent/congruent) × action (approach/avoid) × group (high-anxious/non-anxious) as variables was conducted to examine the group difference on behavioral performance during action control. In addition, for response reaction time on the correct Go trials (e.g. approach trials) a two-way mixed ANOVA with congruence (incongruent/congruent) × group (high-anxious/non-anxious) as variables was conducted. Furthermore, group differences on the behavioral congruency-effect were conducted with independent samples t-tests. In detail, the behavioral congruency-effect was calculated as the response accuracy in the congruent condition (approaching happy stimuli and avoiding fearful stimuli) minus the incongruent condition (approaching fearful stimuli and avoiding happy stimuli). All these data analyses were performed with SPSS Version 25.0 (IBM Corp., Armonk, NY, USA). To further test the null hypotheses that there were no group differences on the behavioral congruency-effect as Bramson *et al*. (2023) reported, Bayesian independent t-tests were conducted using JASP 0.17.1 (https://jasp-stats.org).

#### MRI data acquisition

Neuroimaging data were collected using a 3T GE Discovery MR750 system (General Electric Medical System, Milwaukee, WI, USA) and a total of 488 volumes of T2*-weighted echo planar images were acquired. Details of acquisition parameters are given in the Supplementary Information.

#### MRI data analysis

##### fMRI data preprocessing

Functional MRI data were preprocessed using SPM12 software (Wellcome Trust Centre of Neuroimaging, University College London, London, UK). The first 10 volumes for each run were deleted to allow magnet steady data. The remaining functional images were processed using the standard preprocessing procedures (see Supplementary Information).

##### fMRI data analysis

The general linear model (GLM) was conducted for the first-level analyses using an event-related analysis approach. Six condition-specific regressors [Happy Go (HG, i.e. approaching-congruent), Happy NoGo (HNG, i.e. avoiding-incongruent), Fearful Go (FG, i.e. approaching-incongruent), Fearful NoGo (FNG, i.e. avoiding-congruent), Neutral Go (NeuG), Neutral NoGo (NeuNG)] were modeled on the first level and convolved with the standard hemodynamic response function (HRF). To further control for head movement-related artifacts the six head-motion parameters were included as nuisance regressors. Then, contrast images of interest [neural congruency-effect: (HNG + FG) > (HG + FNG); emotional context-specific contrasts: HNG > HG; FG > FNG)] were created on the individual level. In the second-level model analysis, to examine the general neural-congruent effect across and within groups as well as between-group differences on it, one-sample t-tests and two-sample independent t-tests on (HNG + FG) > (HG + FNG) contrasts were computed respectively. In addition, to examine the context-specific neural mechanism underlying action control as well as group differences on it, one-sample t-tests and two-sample independent t-tests on emotion-specific contrasts (HNG > HG; FG > FNG) were computed respectively.

##### Primary analysis: regions of interest (ROI) analyses

The main aim of the current study was to validate the role of a functional shift from the FPl to the DLPFC in anxiety as previously reported by Bramson *et al*. (2023). To this end we re-analyzed data from a large sample of individuals undergoing an fMRI scan. To allow a more sensitive examination of the reported neural-congruence effect on FPl and DLPFC, ROI analyses were conducted as the primary data analyses with small volume correction (SVC, initial thresholding: *P* < 0.001, uncorrected; peak-level, *P* < 0.05, family wise error, FWE) using the 15 mm spheres as masks centered at the coordinates reported by Bramson *et al*. (2023). The primary analyses were followed-up by secondary analyses focusing on (i) extracted parameter estimates in combination with Bayesian analyses to validate the effects or absence of effects, respectively; and (ii) examining associations on the network level using functional connectivity analyses.

##### Secondary analysis 1: parameter estimate-based analyses and Bayesian analyses

To future determine the association between trait anxiety and brain activity in the FPl and DLPFC, parameter estimate-based analyses and Bayesian analyses were employed as the secondary data analyses in the current study. First, to explore the group differences on the brain activity of FPl and DLPFC, parameter estimates were extracted as mean values of voxels within the regions in each condition using the spm_select and spm_summarise functions implemented in Matlab (R2018a). In addition, brain and trait association analyses including LSAS and total STAI scores were performed with age and gender as covariates, in which one-tailed significance tests (*P* < 0.05) were set (based on previous findings showing a directed shift with increasing anxiety levels, Bramson *et al*., 2023). Furthermore, to further validate the absence of FPl recruitment in high-anxious individuals as well as the absence of DLFPC in the non-anxious individuals, Bayesian independent t-tests were performed using JASP 0.17.1 (https://jasp-stats.org).

##### Secondary analysis 2: functional connectivity analyses

To determine whether the connectivity patterns between sgACC and lateral PFC subregions, i.e. DLPFC and FPl, would be shifted as a function of different anxiety levels, especially in the negative context, functional connectivity analyses were conducted in each group using the generalized psychophysiological interaction analyses (gPPI) approach (McLaren *et al*., 2012) implemented in the CONN toolbox with sgACC as seeds in the positive and negative context separately. The seeds were defined as 6 mm spheres centered at the peak coordinates of sgACC activation mapping (within the sgACC mask from the Human brainnetome atlas; Fan *et al*., 2016) from the negative (contrast: FG > FNG) and positive condition (contrast: HNG > HG) across groups (for details of sgACC activation in different emotional contrasts see Table S3). The definition for regressors during the gPPI analyses included psychophysiological interactions for the HG condition (i.e. approaching-congruent), HNG condition (i.e. avoiding-incongruent), FG condition (i.e. approaching-incongruent), FNG condition (i.e. avoiding-congruent), NeuG and NeuNG condition, task regressors for the above six conditions, seed region timecourse (i.e. left or right sgACC), and a constant, which allows the testing of any combination of all conditions (McLaren *et al*., 2012).

In addition, parameter estimates were extracted as mean values of voxels within the identified regions in each condition for both sgACC–bilateral FPl and sgACC–bilateral DLPFC functional connectivity. Then, analyses of the functional connectivity strength and scales association including LSAS and total STAI scores were performed with age and gender as covariates respectively.

##### Thresholding

For the ROI analysis at the blood oxygen level-dependent (BOLD) level, SVC was performed with the initial thresholding: *P* < 0.001 (uncorrected) and peak-level, *P*_FWE _< 0.05. In addition, for analysis of sgACC–lateral prefrontal region-focused functional connectivity, SVC (initial thresholding: *P* < 0.001, uncorrected; peak-level, *P*_FWE _< 0.05) was employed using the 15 mm spheres as masks, centered at the coordinates reported by Bramson *et al*. (2023). For the brain and scales association analyses, correction for multiple comparisons with respect to brain activity (*P* < 0.05/4 = 0.0125) and functional connectivity (*P* < 0.05/8 = 0.006) was conducted respectively.

## Results

### Behavioral results

Mixed ANOVA on behavioral response accuracy showed no significant interaction effect (*F*_(1,225) _= 0.001, *P* = 0.970) but a main effect of action (*F*_(1 225) _= 692.071, *P* < 0.001) and congruence (*F*_(1,225) _= 6.174, *P* = 0.014, Fig. 1B, for details see Supplementary Information), which was in line with the findings from Bramson *et al*. (2023). Additionally, in line with Bramson *et al*. (2023), the difference between high-anxiety and non-anxious group on the behavioral congruency-effect was not significant (*F*_(1,225) _= 0.415, *P* = 0.520) with a Bayesian t-test showing moderate evidence for the null hypothesis (BF_01 _= 5.634, Fig. 1F). For the response reaction time on the correct approach trials (e.g. Go trials), the mixed ANOVA showed a significant interaction effect between group and congruence (*F*_(1,225) _= 4.213, *P* = 0.041) and a significant main effect of congruence (*F*_(1,225) _= 21.530, *P* < 0.001, see details in the Supplementary Information).

### fMRI results

#### Primary results: ROI analyses

The ROI analyses found a significant neural congruency-effect in the left DLPFC (SVC, *Z* = 3.79, *P*_FWE _= 0.015, voxels = 40, *x*/*y*/*z*: −21, 24, 33, Fig. 1G) across groups rather than FPl for the neural congruency-effect across groups [contrast: (HNG + FG) > (HG + FNG)]. In line with Bramson *et al*. (2023), examination of the anxiety groups separately based on the within-group analyses confirmed that the non-anxious group engaged the left FPl (SVC, *Z* = 3.54, *P*_FWE _= 0.039, voxels = 12, *x*/*y*/*z*: −30, 60, −3. Fig. 1H), while the high-anxious group specifically recruited the DLPFC (SVC, *Z* = 3.47, *P*_FWE _= 0.048, voxels = 13, *x*/*y*/*z*: −27, 42, 36. Fig. 1K). However, no significant between-group differences on the neural level were found.

#### Secondary results 1: parameter-based analyses and Bayesian analyses

The further parameter-based independent t-tests showed significant decreased left FPl activation but increased ipsilateral DLPFC in the high-anxious group than the non-anxious group (Fig. 1I, L), which supports the previous findings of the functional shift between FPl and DLPFC as the anxiety level increased. Additionally, Bayesian t-tests resulted in moderate evidence for the absence of left FPl activation in the high-anxious group (BF_01 _= 9.118, Fig. 1K) as well as the left DLPFC in the non-anxious group (BF_01 _= 8.261, Fig. 1H) during emotional behavior control. Furthermore, the brain and scales association analysis showed that the level of trait social anxiety in the entire sample was significantly positively related with DLPFC activity (*r* = 0.145, *P* = 0.015, Fig. 1M) and negatively with left FPl activation (*r* = 0.138, *P* = 0.019, Fig. 1J) across groups (following a Bonferroni correction *P* < 0.05/4 = 0.0125, trend-to-significant) but not with STAI scores (all *P* > 0.226) as reported by Bramson *et al.* (2023).

#### Secondary results 2: functional connectivity analysis

**Figure 2:**
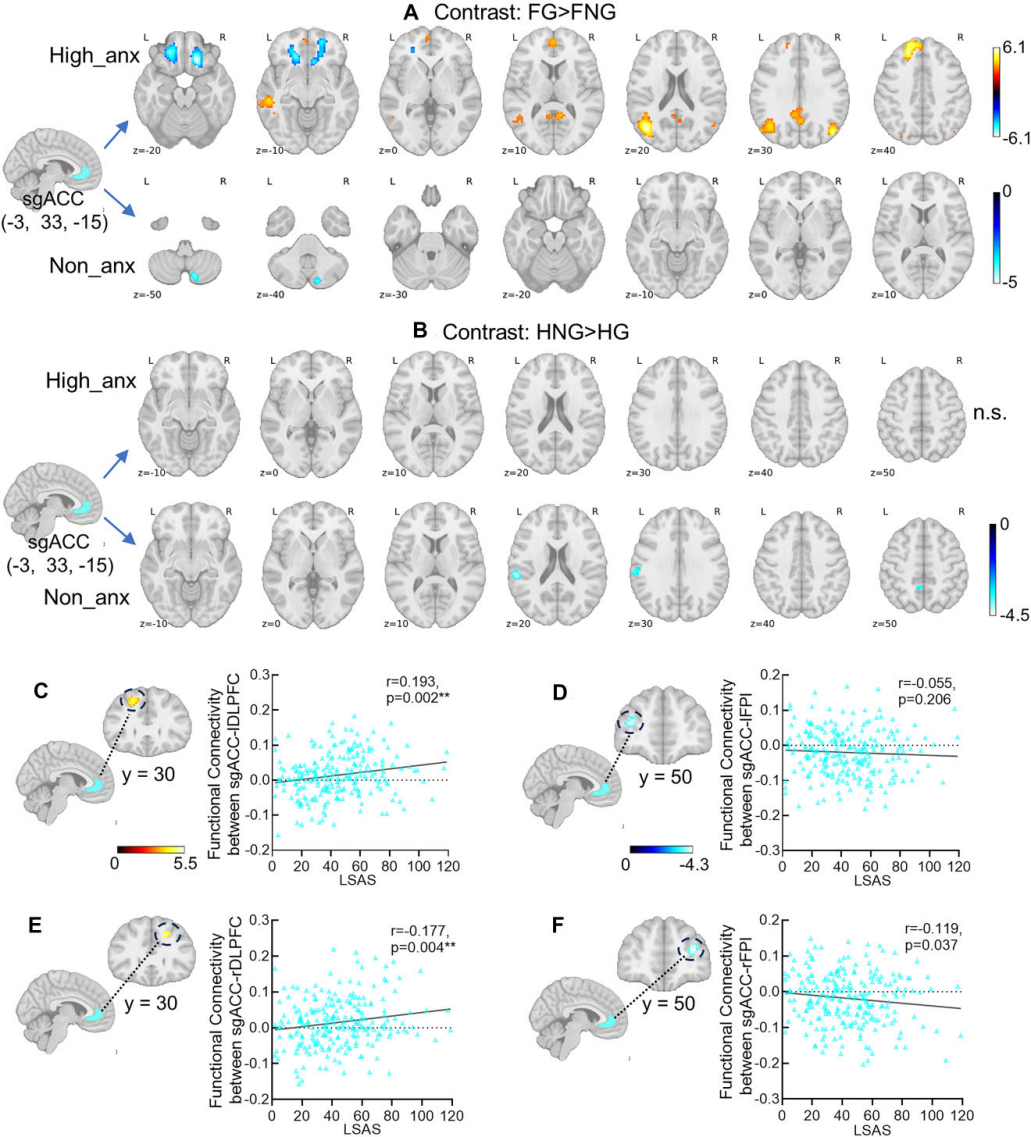
Functional connectivity results in positive and negative emotional context. (A) The results revealed a significant positive connectivity between left sgACC (seed defined by the contrast: FG > FNG) and bilateral DLPFC as well as a significant negative connectivity with bilateral FPl for the high-anxiety group rather than the non-anxious group especially in the negative context after small volume correction with corresponding masks centered into the peak coordinates reported by Bramson *et al*. (2023). (B) No significant connectivity between sgACC (seed defined by the contrast: FG > FNG) and lateral frontal areas was found in the positive context either for the high-anxiety or non-anxious group. The brain and scales association analyses across groups showed significant positive correlations between LSAS scores with sgACC–bilateral DLPFC connectivity strength in the negative context (C: sgACC–left DLPFC, *r* = 0.193, *P* = 0.002; E: sgACC–right DLPFC, *r* = 0.177, *P* = 0.004) but not with the sgACC–bilateral FPI connectivity strength after the correction for multiple comparisons (D: sgACC–left FPl, *r* = 0.055, *P* = 0.206; F: sgACC–right FPl, *r* = 0.119, *P* = 0.037). Note: DLPFC, dorsolateral prefrontal cortex; FPl, lateral frontal pole; High-anx: high-anxious group; non-anx: non-anxious group; sgACC, subgenual anterior cingulate cortex. **P < 0.01.

Examining the emotional context-dependent (contrasts: FG > FNG; HNG > HG) functional connectivity patterns in the non- and high-anxious group separately and focusing on the sgACC–FPl and sgACC–DLPFC circuitry, we found a significant positive connectivity between left sgACC (seed defined from the contrast: FG > FNG) and bilateral DLPFC as well as a negative connectivity with bilateral FPl for the high-anxious group specific to the negative context (Fig. 2A, for results in the positive context see Fig. 2B) after SVC (initial thresholding: *P* < 0.001, uncorrected; SVC: left DLPFC, *Z* = 5.22, *P*_FWE _< 0.001, voxels = 96, *x*/*y*/*z*: −21, 39, 39, Fig. 2C; right DLPFC, *Z* = 3.79, *P*_FWE _= 0.031, voxels = 28, *x*/*y*/*z*: 18, 30, 39, Fig. 2E; left FPl, *Z* = 4.16, *P*_FWE _= 0.008, voxels = 53, *x*/*y*/*z*: −33, 51, 12, Fig. 2D; right FPl, *Z* = 4.12, *P*_FWE _= 0.016, voxels = 37, *x*/*y*/*z*: 33, 51, 15, Fig. 2F), potentially reflecting network-level markers for increased social anxiety. Additionally, across both groups the level of social anxiety was positively associated with sgACC–bilateral DLPFC connectivity strength (sgACC–left DLPFC: *r* = 0.193, *P* = 0.002, Fig. 2C; sgACC–right DLPFC: *r* = 0.177, *P* = 0.004, Fig. 2E) but not with sgACC–FPl connectivity after the multiple comparison corrections (sgACC–left FPl: *r *= 0.055, *P* = 0.206, Fig. 2D; sgACC–right FPl: *r* = 0.119, *P* = 0.037, Fig. 2F). No significant correlations between STAI scores and the sgACC–FPI and sgACC–DLPFC connectivity were found (*P* ≥ 0.016). For the sgACC seed from the contrast: HNG > HG, there was a significant positive connectivity with bilateral DLPFC, but not FPl after SVC, specifically for the high-anxious group in the negative context (see Supporting Information). Together these findings may reflect a negative context-specific neural shift in terms of functional connectivity from the sgACC–FPl to sgACC–DLPFC in high social anxiety during emotional action control.

## Discussion

The present study employed a different paradigm in a comparably large sample from a Chinese population and demonstrated a partial replication of the previous findings supporting a functional shift between the FPl and DLPFC during emotional action control in elevated (social) anxiety (as described by Bramson *et al*., 2023). In line with Bramson *et al*. (2023), no significant group differences on the behavioral congruency-effect were observed, which confirmed similar behavioral patterns during emotional action control between the non-anxious and high-anxiety group. On the neural level, while our primary results showed significant FPl recruitment in the non-anxious group and DLPFC recruitment in the high-anxious group based on the within-group analyses, a direct comparison in the present data did not reveal significant neural differences between the groups as Bramson *et al*. (2023) reported.

While our results of the non-significant between-group findings do not replicate the previous findings, further results from the secondary parameter-based analyses and Bayesian analyses support the conclusions drawn by Bramson *et al*. (2023). For instance, (i) parameter-based analyses revealed significantly decreased left FPl activation but increased ipsilateral DLPFC activation in the high-anxiety group as compared to the non-anxious group; (ii) similar with the previous findings (Bramson *et al*., 2023), our results showed a significant positive correlation between the level of trait anxiety with left DLPFC activity but a negative correlation with the left FPl across groups—the opposite correlation patterns underscore an increasing engagement of the DLPFC (as opposed to FPl) during emotional action control when social anxiety symptoms increased; and (iii) further Bayesian analyses revealed moderate evidence for the absence of left FPl activation in the high-anxious group (BF_01 _= 9.118) as well as the left DLPFC in the non-anxious group (BF_01 _= 8.261) during emotional behavior control. Thus, together the results from both behavioral and neural respects suggest that the present findings represent a partial replication of those findings from Bramson *et al*. (2023) (possibly due to the higher sensitivity of dimensional as compared to categorical analyses, see e.g. Chen *et al*., 2023). The discrepancy may possibly be due to the different task paradigms or stimuli used, participants from different cultural backgrounds, or different distributions of trait anxiety in the two studies.

Additionally, our secondary results showed a significant negative context-specific positive sgACC–bilateral DLPFC connectivity and negative sgACC–bilateral FPl connectivity in the high-anxious group. In line with this, previous studies have indicated the important role of vmPFC/ACC-FPl and sgACC-DLPFC connectivity in emotional regulation and their potential role in target-based non-invasive intervention for affective disorders (Fonzo *et al*., 2017; Wang *et al*., 2019; Whitfield-Gabrieli *et al*., 2020; Schiena *et al*., 2021). Together, our findings may reflect that context-specific changes in the communication with the sgACC may underpin deficient emotion regulation in high anxiety.

There are several limitations in the current study. First, Bramson *et al*. (2023) showed a greater GABA/glutamate ratio as well as stronger amygdalofugal projections in the FPl for the high-anxiety group, and given the lack of corresponding data in the present sample we were unable to examine whether the partial replication extends to these findings. Future studies could explore whether the neurochemical and structural changes could be replicated as a function of anxiety level and across cultures. Second, threat-associated negative words rather than other negative stimuli such as sad words were employed in the present paradigm. Whether other negative stimuli such as sad words have a distinct influence on action control could be investigated in future studies.

## Conclusion

The current study using an affective Go/NoGo paradigm partially confirmed a functional–anatomical shift from FPl to DLPFC during emotion regulation in anxiety. Additional analyses suggest a network-level shift involving sgACC connectivity, which shows a progressive shift towards the lateral PFC with the increased social anxiety levels. Together, the findings identify new intervention targets for the treatment of anxious individuals.

## Supplementary Material

kkaf009_Supplemental_File
