# Supplementary material for: A functional anatomical shift from the lateral frontal pole to dorsolateral prefrontal cortex in emotion action control underpins elevated levels of anxiety: partial replication and generalization of Bramson et al., 2023
Source: Psychoradiology. 2025 Apr 28;5:kkaf009. doi: 10.1093/psyrad/kkaf009 (PMC12093096; doi:10.1093/psyrad/kkaf009)
Supplement: kkaf009_Supplemental_File [file kkaf009_supplemental_file.docx]

**Supplementary Information for Review**

A functional anatomical shift from the lateral frontal pole to dorsolateral prefrontal cortex in emotion action control underpins elevated levels of anxiety – partial replication and generalization of Bramson et al., 2023

Zhuang., et al.,

Contact: [bbecker@hku.hk;](mailto:bbecker@hku.hk;)

1. Method

1.1 Task setting

All participants performed an affective Go/NoGo fMRI task with positive (happy), negative (fearful) and neutral words (word length=4, words frequency matched) were used as task stimuli. For example, while the words included Huan Tian Xi Di (欢天喜地) and Xi Chu Wang Wai (喜出望外) were used on the positive condition, Xin Jing Rou Tiao (心惊肉跳) and Ti Xin Diao Dan (提心吊胆) were used on the negative condition and Che Lai Che Wang (车来车往) and Xin Wen Bo Bao (新闻播报) were used on the neutral condition respectively. Participants were instructed to respond according to the font of the words regardless of the meaning, such as, pressing the button when the words were presented as normal font (i.e. go trials, approach condition), while withdrawing the response when the words were occasionally in italic font (i.e. nogo trials, avoid condition).

During the data quality control, n=23 participants were excluded due to technical issues or excessive head motion during data acquisition. In detail, data from 11 subjects were lost due to technical issues during fMRI (n=5) and behavioral data collection (n=6) and data from another 12 subjects were further excluded due to excessive head motion (>2.5mm or 2.5 degrees, n=12). Thus, there are n = 227 subjects were included in the final behavioral and fMRI data analyses (112 males; age: mean ± SD=21.62 ± 2.32 years) in which n=9 subjects with only first run data. All participants were divided into high-anxious (n=127, 64 female, age: mean+SEM=21.73+0.20) and non-anxious (n=100, 51 female, age: mean+SEM=21.48+0.24) group according to the validated Liebowitz Social Anxiety Scale (LSAS, Mennin et al., 2002; subscale fear: Cronbach’a =0.937; avoid: Cronbach’a=0.917) with 38 as the cutoff score. There was a significant group difference on the independent measurement of both trait (high-anx: mean+SEM=44.49+0.83; non-anx: mean+SEM=39.05+0.93, t_(225)_=4.37, p<0.001) and state anxiety (high-anx: mean+SEM=41.94+0.86; non-anx: mean+SEM=37.55+0.93, t_(225)_=3.44, p<0.001) scores assessed by State Trait Anxiety Inventory (STAI, Spielberger et al., 1983).

1.2 Data acquisition and analyzing

1.2.1 MRI Data Acquisition

Neuroimaging data were collected using a 3T GE Discovery MR750 system (General Electric Medical System, Milwaukee, WI). A total of 488 volumes of T2*-weighted echo planar images were acquired (acquisition parameters: repetition time, 2000ms; echo time, 30 ms; slices, 39; slice-thickness, 3.4mm; gap, 0.6mm; field of view, 240 × 240 mm^2^; matrix size, 64 × 64; flip angle, 90°). To improve normalization of the functional images and identify individuals with apparent brain pathologies, high-resolution whole brain T1-weighted images were obtained using a 3D spoiled gradient echo pulse sequence (acquisition parameters: repetition time, 6ms; echo time, minimum; flip angle, 9°; field of view = 256 × 256mm; acquisition matrix, 256 × 256; thickness, 1mm; 156 slices). OptoActive MRI headphones (http://www.optoacoustics.com/) were used to reduce acoustic noise during MRI data acquisition.

1.2.2 fMRI Data Preprocessing

Functional MRI data were preprocessed using SPM12 software (Wellcome Trust Center of Neuroimaging, University College London, London, United Kingdom). The first 10 volumes for each run were deleted to allow magnet steady data. The remaining functional images were processed using the following standard preprocessing procedures: (1) slice-timing and head motion correction, (2) spatial normalization to Montreal Neurological Institute (MNI) standard space (by means of co-registration to the T1-weighted structural images and the application of the transformation matrices obtained from the segmentation of the structural images), (3) resampling with a 3 x 3 x 3 mm resolution, and (4) spatial smoothing using a 8mm full-width at half-maximum (FWHM) Gaussian kernel.

1. Results

3.1 Behavioral results

For the response accuracy, the mixed ANOVA analysis with congruence (incongruent/congruent) *action (approach/avoid)*group (high-anxious/non-anxious) as variables showed a main effect of congruence (F_(1,225)_=6.174, p=0.014) and action (F_(1,225)_=692.071, p<0.001), with a significantly decreased accuracy in the incongruent condition compared to the congruent condition (incongruent: mean+SEM=84%+0.70; congruent: mean+SEM=85%+0.60) and an increased accuracy on the approaching condition compared to avoiding condition (approach: mean+SEM=98.5%+0.20; avoid: mean+SEM=70.5%+1.10). No significant interaction effect between congruence*action*group was found (F_(1,225)_=0.001, p=0.970).

For the response reaction time on the correct approach trials (e.g. Go trials), the mixed ANOVA analysis showed a significant interaction effect between group and congruence (F_(1,225)_=4.213, p=0.041), with the further Post-hoc tests showing that the reaction time in the incongruent condition was significantly higher than congruent condition for non-anxious group (incongruent: mean+SEM=322.127ms+5.856; congruent: mean+SEM= 314.456ms+5.604; p<0.001) but not the high-anxious group (incongruent: mean+SEM=320.988ms+5.196; congruent: mean+SEM=318.022ms+4.973; p=0.053, Fig.S1). This might indicate an over-generalized response when approaching positive stimuli and negative stimuli for the high-anxious group. In addition, in line with the findings reported by Bramson et al., (2023), the result also found a significant main effect of congruence (F_(1,225)_=21.530, p<0.001), with the reaction time in the incongruent condition was significantly higher than congruent condition (incongruent: mean+SEM=321.557ms+3.914; congruent: mean+SEM= 316.239ms+3.746).

3.2 fMRI results

3.2.1 BOLD level results

The whole brain cluster-level correction was employed on both BOLD level and functional connectivity analyses (initial thresholding: p<0.001, cluster-level, pFWE<0.05, see Supplementary Information). For the across groups neural congruency-effect, the results showed a significant activation in left MOG (middle occipital gyrus, MOG) extending to angular and SPG (superior parietal gyrus, SPG) on the whole brain level (initial thresholding p<0.001 uncorrected, cluster level, p_FWE_<0.05, see Table. S2).

Context-dependent activation analyses showed that both high-anxious and non-anxious groups showed similar activation patterns in FPl and DLPFC after SVC in different emotional contexts (see Table S3) and widespread brain activation in regions located at fronto-parietal, occipital and temporal lopes on the whole brain level (initial thresholding: p<0.001 uncorrected; cluster level, p_FWE_<0.05, see Fig. S2). In addition, no significant between-group differences were found in neither the negative (contrast: FG>FNG) nor positive context (contrasts: HNG>HG).

3.3 Functional connectivity results

Functional connectivity analyses were performed in the positive and negative context respectively with sgACC seeds defined from either positive or negative emotional action control (contrasts: HNG>HG; FG>FNG). For the sgACC seed defined from the positive contrast (HNG>HG), the results showed a positive connectivity between right sgACC and bilateral DLPFC but not FPl after SVC especially in the negative condition for high-anxious group (SVC: left DLPFC, Z=3.84, p_FWE_ =0.025, voxels=15, x/y/z: -24, 42, 27; right DLPFC, Z=4.06, p_FWE_ =0.012, voxels=23, x/y/z: 30, 42, 30). On the whole brain level, we found a positive connectivity between right sgACC and right ITG for high-anxious group in the negative context (Fig.S3A, details see Table. S4). In addition, while in the positive context there was a positive connectivity between right sgACC with ipsilateral caudal regions and left IFG and a negative connectivity with bilateral precuneus and left postcentral cortex for high-anxious group, the results showed a positive connectivity between right sgACC and fusiform, left MFG and a negative connectivity with bilateral precuneus for non-anxious group (Fig.S3B, details see Table. S4).

For the sgACC seed from the negative contrast (FG>FNG), the results showed a significant positive connectivity between left sgACC and bilateral DLPFC as well as a significant negative connectivity with bilateral FPl for the high-anxious group rather than the non-anxious group especially in the negative context after SVC. On the whole brain level while the results found a significant positive sgACC connectivity with left SFG, mOFC, MTG, MOG, right precuneus and angular regions and negative connectivity with bilateral mOFC and right cerebellum for high-anxious group, there is a negative connectivity between sgACC and right cerebellum for non-anxious group in the negative context (cluster level, p_FWE_<0.05, details see Table. S5).

3.4 Brain and scales association results

After the correction for multiple comparisons (p<0.05/8=0.006), the results showed significant positive correlations between LSAS scores with sgACC-bilateral DLPFC connectivity strength in the negative context (sgACC-left DLPFC: r=0.193, p=0.002; sgACC-right DLPFC: r=0.177, p=0.004) but not with the sgACC-bilateral FPI connectivity strength (sgACC-left FPl: r=0.055, p=0.206; sgACC-right FPl: r=0.119, p=0.037). In addition, no significant correlations between STAI scores and the sgACC-FPI and sgACC-DLPFC connectivity were found (ps≥0.016).

**References**

Bramson, B., Meijer, S., van Nuland, A., et al. (2023) Anxious individuals shift emotion control from lateral frontal pole to dorsolateral prefrontal cortex. Nat Commun 14(1): 4880.

Fan L, Li H, Zhuo J, et al. (2016) The human brainnetome atlas: a new brain atlas based on connectional architecture. Cereb Cortex 26(8): 3508-3526.

Mennin, D. S., Fresco, D. M., Heimberg, R. G., et al. (2002) Screening for social anxiety disorder in the clinical setting: using the Liebowitz Social Anxiety Scale. J. Anxiety. Disord 16(6): 661-673.

Spielberger, C. D., Gorsuch, R. L., Lushene, R. E., et al. (1983). Manual for the state-trait anxiety inventory. Mindgarden, San Diego, CA.


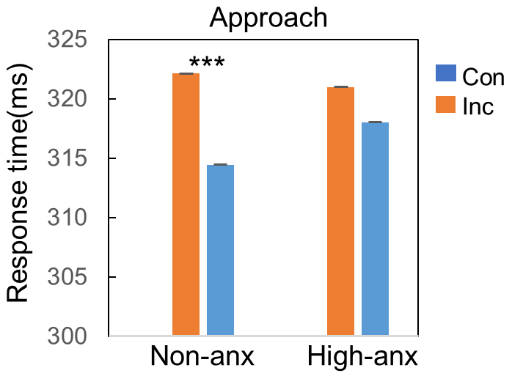


**Figure legend**

Fig. S1 The congruent effect on the response time for the correct approach trials (e.g. Go trials). The mixed ANOVA analysis showed a significant interaction effect between group and congruence (F_(1,225)_=4.213, p=0.041), with the further Post-hoc tests showing that the reaction time in the incongruent condition was significantly higher than congruent condition for non-anxious group (incongruent: mean+SEM=322.127ms+5.856; congruent: mean+SEM=314.456ms+5.604; p<0.001) but not the high-anxious group (incongruent: mean+SEM=320.988ms+5.196; congruent: mean+SEM=318.022ms+4.973; p=0.053). In addition, the result also found a significant main effect of congruence (F_(1,225)_=21.530, p<0.001), with the reaction time in the incongruent condition was significantly increased than congruent condition (incongruent: mean+SEM=321.557ms+3.914; congruent: mean+SEM=316.239ms+3.746). Note: Con: congruent; High-anx: high-social anxious group; Inc: incongruent; Non-anx: Non-social anxious group.*** means p<0.001.


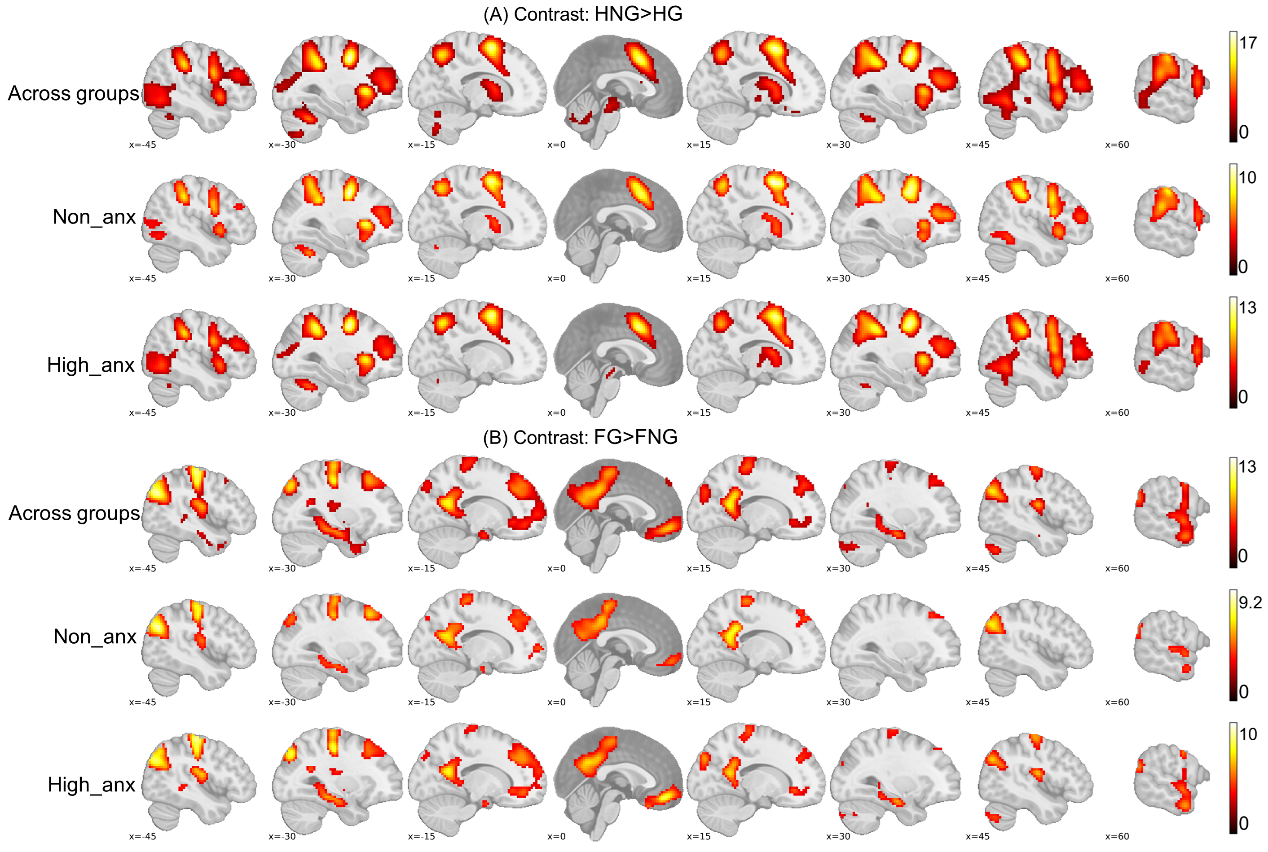


Fig. S2 Context-dependent activation mapping during action control on the whole brain level. (A) Brain activation mapping of action control in the positive context (contrast: HNG>HG) across groups as well as for both high-anxious and non-anxious group. (B) Brain activation mapping of action control in the negative context (contrast: FG>FNG) across groups as well as for both high-anxious and non-anxious group. All clusters passed the initial thresholding p<0.001 uncorrected, then cluster level p_FWE_ < 0.05. FWE, Family Wise Error. Note: High-anx: high-social anxious group; Non-anx: Non-social anxious group.


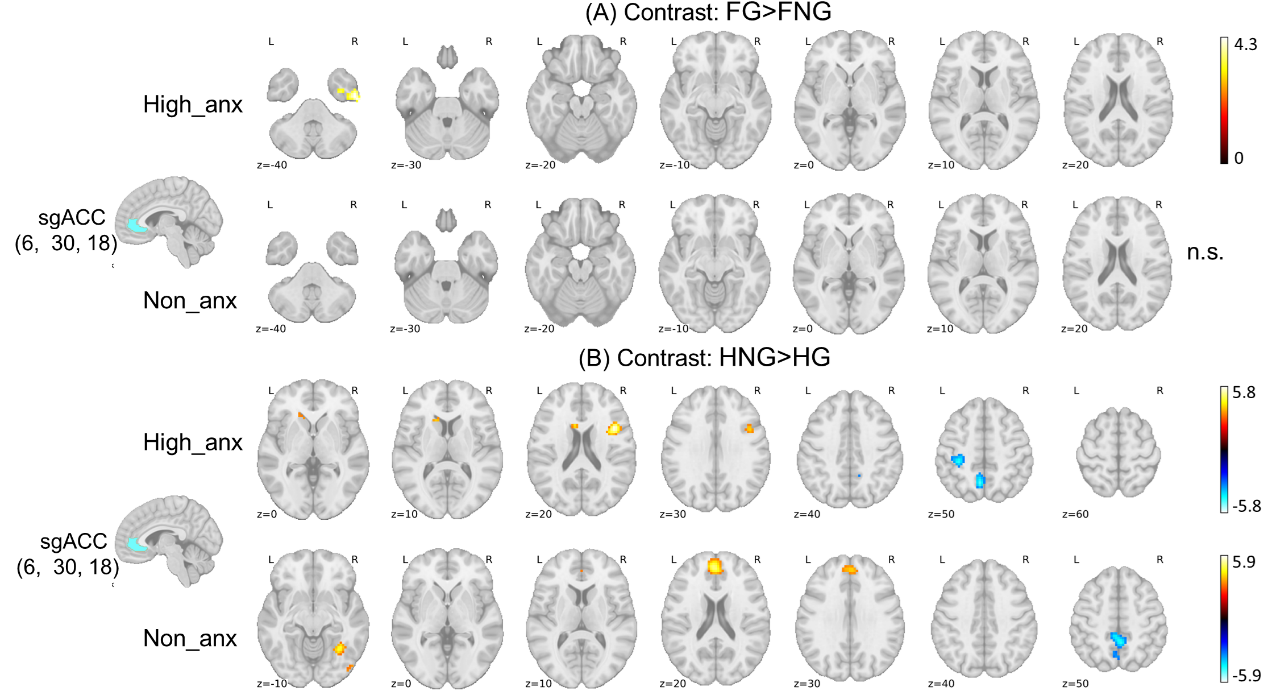


Fig. S3 Functional connectivity results for sgACC (seed defined from the contrast: HNG>HG) in the negative and positive context. (A)The results showed a positive connectivity between right sgACC and bilateral DLPFC especially in the negative condition for high-anxious group after SVC (SVC: left DLPFC, Z=3.84, p_FWE_ =0.025, voxels=15, x/y/z: -24, 42, 27; right DLPFC, Z=4.06, p_FWE_ =0.012, voxels=23, x/y/z: 30, 42, 30). On the whole brain level, the results showed a positive connectivity between right sgACC and right ITG for high-anxious group in the negative context. (B) While in the positive context there was positive connectivity between right sgACC with ipsilateral caudal regions and left IFG and a negative connectivity with bilateral precuneus and left postcentral cortex for high-anxious group, the results showed a positive connectivity between right sgACC and fusiform, left MFG and a negative connectivity with bilateral precuneus for non-anxious group. Note: High-anx: high-social anxious group; Non-anx: Non-social anxious group.

**Tables**

**Table S1. sgACC activation across groups during action control in either positive or negative contexts (contrasts: HNG>HG; FG>FNG).**

| Regions | Cluster K | Coordinates | | | t value |
| --- | --- | --- | --- | --- | --- |
|  |  | X | Y | Z |  |
| contrast: HNG>HG |  |  |  |  |  |
| r sgACC | 10 | 6 | 30 | 18 | 6.67 |
|  |  | 9 | 36 | 15 | 4.98 |
| contrast: FG>FNG |  |  |  |  |  |
| l sgACC | 35 | -3 | 45 | -9 | 4.93 |
|  |  | -3 | 33 | -15 | 4.68 |
|  |  | -6 | -39 | -12 | 4.81 |

Note: Threshold: initial thresholding p<0.001 uncorrected, then small volume correction with the sgACC mask from the Human brainnetome atlas (Fan et al., 2016), peak level p_FWE_ < 0.05. FWE, Family Wise Error; l, left; r, right; sgACC, subgenual anterior cingulate cortex.

**Table S2. The neural congruency-effect across groups on the whole brain level.**

| Regions | Cluster K | Coordinates | | | t value |
| --- | --- | --- | --- | --- | --- |
|  |  | X | Y | Z |  |
| l MOG extending to angular and SPG | 247 | -36 | -78 | 36 | 4.54 |
|  |  | -39 | -60 | 30 | 3.62 |
|  |  | -18 | -75 | 45 | 3.31 |

Note: All clusters passed the thresholding at: initial thresholding p<0.001 uncorrected, cluster level p_FWE_ < 0.05. FWE, Family Wise Error; l, left; MOG, middle occipital gyrus; SPG, superior parietal gyrus.

**Table S3. FPl and DLPFC activation in each group during behavioral control in either positive or negative context (contrasts: HNG>HG; FG>FNG).**

| Regions | Cluster K | Coordinates | | | t value |
| --- | --- | --- | --- | --- | --- |
|  |  | X | Y | Z |  |
| High-anxious group |  |  |  |  |  |
| Contrast: HNG>HG |  |  |  |  |  |
| l FPl | 140 | -30 | 48 | 12 | 6.37 |
| r FPl | 170 | 33 | 45 | 15 | 6.16 |
| l DLPFC | 57 | -30 | 39 | 27 | 5.18 |
| r DLPFC | 75 | 36 | 36 | 27 | 7.14 |
| Contrast: FG>FNG |  |  |  |  |  |
| l FPl | 32 | -21 | 57 | 0 | 4.19 |
| l DLPFC | 260 | -21 | 27 | 39 | 7.30 |
| r DLPFC | 94 | 18 | 39 | 36 | 4.80 |
| Non-anxious group |  |  |  |  |  |
| Contrast: HNG>HG |  |  |  |  |  |
| l FPl | 112 | -33 | 45 | 12 | 4.95 |
| r FPl | 150 | 27 | 48 | 15 | 6.37 |
| l DLPFC | 36 | -30 | 36 | 21 | 4.62 |
| r DLPFC | 54 | 33 | 36 | 24 | 6.12 |
| Contrast: FG>FNG |  |  |  |  |  |
| l FPl | 18 | -18 | 63 | 3 | 4.33 |
| l DLPFC | 224 | -24 | 27 | 48 | 6.58 |
| r DLPFC | 110 | 21 | 42 | 42 | 5.49 |

Note: Threshold: p<0.001 uncorrected, then small volume correction using the 15mm spheres as masks which is centered at the coordinates reported by Bramson et al., (2023), peak level p_FWE_ < 0.05. DLPFC, dorsolateral prefrontal cortex; FPl, lateral frontopolar cortex; FWE, Family Wise Error; l, left; r, right.

**Table S4. Right sgACC (seed defined from contrast: HNG>HG) connectivity results for high-anxious and non-anxious group on the whole brain level.**

| Regions | Cluster K | Coordinates | | | t value |
| --- | --- | --- | --- | --- | --- |
|  |  | X | Y | Z |  |
| Results for high-anxious group in the negative context | | | | | |
| Positive connectivity with sgACC | | | | | |
| r ITG | 108 | 54 | -12 | -39 | 4.34 |
|  |  | 39 | -3 | -42 | 4.28 |
|  |  | 48 | -6 | -48 | 3.89 |
| Results for high-anxious group in the positive context | | | | | |
| Positive connectivity with sgACC | | | | | |
| r IFG | 132 | 42 | 9 | 24 | 5.77 |
|  |  | 45 | 30 | 15 | 3.99 |
| l Caudate | 89 | -12 | 18 | 15 | 4.75 |
|  |  | -30 | 27 | 15 | 4.13 |
|  |  | -24 | 15 | 15 | 3.53 |
| Negative connectivity with sgACC | | | | | |
| l Precuneus | 107 | -6 | -60 | 51 | 4.74 |
|  |  | 9 | -54 | 45 | 4.13 |
| l Postcentral cortex | 85 | -33 | -33 | 51 | 4.50 |
| Results for non-anxious group in the positive context | | | | | |
| Positive connectivity with sgACC | | | | | |
| l MFG | 217 | -3 | 51 | 21 | 5.20 |
| r Fusiform | 125 | 33 | -57 | -6 | 4.83 |
|  |  | 48 | -78 | -6 | 4.14 |
| Negative connectivity with sgACC | | | | | |
| l Precuneus | 336 | 0 | -48 | 57 | 5.95 |
|  |  | -3 | -57 | 60 | 5.55 |

Note: All clusters passed the initial thresholding p<0.001 uncorrected, then cluster level p_FWE_ < 0.05. FWE, Family Wise Error; IFG, inferior frontal gyrus; ITG, inferior temporal gyrus; l, left; MFG, middle frontal gyrus; r, right; sgACC, subgenual Anterior Cingulate Cortex.

**Table S5. Left sgACC (seed defined from contrast: FG>FNG) connectivity results for high-anxious and non-anxious group in the negative context on the whole brain level.**

| Regions | Cluster K | Coordinates | | | t value |
| --- | --- | --- | --- | --- | --- |
|  |  | X | Y | Z |  |
| Results for high-anxious group | | | | | |
| Positive connectivity with sgACC | | | | | |
| l SFG | 283 | -21 | 39 | 39 | 5.53 |
|  |  | -15 | 48 | 39 | 5.38 |
|  |  | -21 | 27 | 45 | 4.62 |
| r mOFC | 139 | 0 | 51 | 6 | 3.99 |
|  |  | 0 | 51 | -15 | 3.98 |
|  |  | -3 | 60 | 3 | 3.82 |
| r Precuneus | 208 | 6 | -54 | 15 | 4.20 |
|  |  | 0 | -60 | 30 | 4.03 |
|  |  | -9 | -51 | 30 | 3.91 |
| l MOG | 408 | -39 | 0 | 3 | 5.46 |
|  |  | -57 | -18 | 33 | 5.29 |
|  |  | -63 | -18 | 21 | 5.21 |
| r Angular | 101 | 48 | -75 | 33 | 5.89 |
|  |  | 57 | -63 | 15 | 4.46 |
| l MTG | 103 | -60 | -33 | -6 | 4.36 |
|  |  | -48 | -48 | -6 | 4.32 |
| Negative connectivity with sgACC | | | | | |
| l mOFC | 288 | 18 | 27 | -21 | 6.07 |
|  |  | 21 | 54 | -9 | 4.83 |
|  |  | 9 | 24 | -12 | 4.69 |
| r mOFC | 265 | -21 | 39 | -21 | 5.31 |
|  |  | -18 | 33 | -9 | 5.17 |
|  |  | -24 | 42 | -6 | 4.85 |
| r Cerebellum | 115 | 33 | -51 | -45 | 5.23 |
|  |  | 24 | -42 | -54 | 4.58 |
| Results for non-anxious group | | | | | |
| Negative connectivity with sgACC | | | | | |
| r Cerebellum | 96 | 15 | -78 | -45 | 5.01 |

Note: All clusters passed the initial thresholding p<0.001 uncorrected, then cluster level p_FWE_ < 0.05. FWE, Family Wise Error; l, left; mOFC, medial orbital frontal cortex; MOG, Middle occipital gyrus; MTG, Middle temporal gyrus; r, right; SFG, Superior frontal gyrus; sgACC, subgenual Anterior Cingulate Cortex.
